# Antidiabetic Effect of Tibetan Medicine Tang-Kang-Fu-San on High-Fat Diet and Streptozotocin-Induced Type 2 Diabetic Rats

**DOI:** 10.1155/2017/7302965

**Published:** 2017-08-17

**Authors:** Bailu Duan, Zhongqiu Zhao, Ling Lin, Jing Jin, Lijun Zhang, Hui Xiong, Na Ta, Tiexiang Gao, Zhinan Mei

**Affiliations:** ^1^College of Basic Medicine, Hubei University of Chinese Medicine, Wuhan 430065, China; ^2^College of Pharmaceutical Sciences, South-Central University for Nationalities, Wuhan 430074, China; ^3^Center for the Study of Itch, Department of Anesthesiology, Washington University School of Medicine, St. Louis, MO 63110, USA; ^4^Barnes-Jewish Hospital, St. Louis, MO 63110, USA; ^5^College of Nursing & Medical Technology, Jianghan University, Wuhan 430056, China; ^6^Department of Encephalopathy, Wuhan Hospital of Traditional Chinese Medicine, Wuhan 430014, China

## Abstract

The aim of this study was to investigate the antidiabetic effects of a Tibetan medicine, Tang-Kang-Fu-San (TKFS), on experimental type 2 diabetes mellitus (T2DM) rats and to explore its underlying mechanisms. Firstly two major chemical compositions of TKFS, gallic acid and curcumin, were characterized by HPLC fingerprint analysis. Next T2DM in rats was induced by high-fat diet and a low-dose streptozotocin (STZ 35 mg/kg). Then oral gavage administration of three different doses of TKFS (0.3 g/kg, 0.6 g/kg, and 1.2 g/kg) was given to T2DM rats. Experimental results showed that TKFS dramatically reduced the levels of fasting blood glucose, fasting blood insulin, triglyceride, total cholesterol, LDL cholesterol, and HDL cholesterol, even though it did not alter the animal body weight. The downregulation of phosphorylation-AKT (p-AKT) and glucose transporter-4 (GLUT4) in skeletal muscle of T2DM rats was restored and abnormal pathological changes in pancreas tissues were also improved. Our work showed that TKFS could alleviate diabetic syndromes, maintain the glucose homeostasis, and protect against insulin resistance in T2DM rats, and the improvement of AKT phosphorylation and GLUT4 translocation in skeletal muscle would be one of its possible underlying mechanisms.

## 1. Introduction

Type 2 diabetes mellitus (T2DM) is a heterogeneous group of metabolic disorders including deranged fat, protein, and carbohydrate metabolism and featured by hyperglycemia resulting from insulin resistance and inadequate insulin secretion [1]. According to the cartographic picture of diabetes provided by the International Diabetes Federation, T2DM has been a public health problem threatening both developed and developing countries and carried over into our next generations [1, 2]. Thus effective prevention and treatment of T2DM will tremendously reduce the medical costs and benefit the public health [1–3]. To date there are several classes of oral antidiabetic medications including biguanides, sulfonylureas, meglitinide, thiazolidinedione, dipeptidyl peptidase 4 inhibitors, sodium-glucose cotransporter inhibitors, and *α*-glucosidase inhibitors [4, 5]. However, due to their existing adverse side effects and increasing costs [4], it is still attracting the global research to seek novel therapies for T2DM.

Experimental animal models provide us with great opportunities to gain information about how different genetic and environmental factors are influencing T2DM and to develop new approaches treating the disease. In the last decades a number of animal models had been developed via different chemical, surgical, or genetic ways to duplicate the clinical features or complications of T2DM with varying degrees of insulin resistance and *β*-cell failure [6]. In fact, due to the complexity of T2DM it is known that none of these animal models can fully represent all diverse syndromes seen in patients [6]. In recent years a nongenetic rat model, *β*-cell-toxicity of streptozotocin (STZ) resulting in *β*-cell necrosis, has been often used to combine with high-fat diet resulting in *β*-cell failure and insulin resistance [6, 7]; therefore, we also chose this model to study the mechanisms and treatments of T2DM.

Traditional Chinese Medicine (TCM) has long been the foundation in the treatment and prevention of many diseases and approximately 800 plants have been identified in the treatment or prevention of T2DM [8]. Because of their reputation for safety, efficacy, and low cost, there is growing interest in research and pharmaceutical industry to study TCM herbs as candidates for the prevention and treatment of T2DM [8, 9]. Tang-Kang-Fu-San (TKFS), a traditional Tibetan medicine and a herbal formula developed under a careful study of “rGyud-bzhi” (a principal textbook of Tibetan medicine), has been used to treat T2DM for many years in China, especially in the Qinghai-Tibet Plateau. However, scientific evidence related to its antidiabetic efficacy and exact mechanisms was still absent. Therefore, to better cope with those challenges we chose combination of high-fat diet and a low-dose STZ-induced T2DM rats as our animal model and studied the antidiabetic effects and the possible intracellular mechanisms of TKFS for treating the T2DM.

## 2. Materials and Methods

### 2.1. HPLC Method for Quantitative Analysis of Gallic Acid and Curcumin

The herbal formula of TKFS was provided by Tibet Autonomous Region Institute of Traditional Tibetan Hospital. High performance liquid chromatography (HPLC) analysis was used to analyze active constituents of gallic acid and curcumin in TKFS. 0.5 g of TKFS powder was extracted with 50 mL of 50% methanol in an ultrasonic water bath (50 : 50, v/v of methanol/water) for 20 min. Gallic acid and curcumin were purchased from Chengdu Must Biological Technology Co., Ltd. (Chengdu, China). Their purities were more than 98% and stock solutions were prepared by dissolving in methanol.

The HPLC fingerprint study as well as the quantitative and qualitative analysis had been performed similarly to previous studies [10, 11]. Shortly, the solvent was filtered through a 0.22 *μ*m pore size microfiltration membrane (Shanghai, China). Next samples were processed via an Agilent 1260 HPLC system (Karlsruhe, Germany), including a quaternary solvent delivery system, an on-line degasser, an autosampler, a column temperature controller, and a photodiode array detector coupled with an analytical workstation. Samples were analyzed through Waters SunFire C18 column (250 mm × 4.6 mm, 5 *μ*m) at 30°C. The binary gradient elution system contained methanol and 0.2% phosphoric acid aqueous solution, and separation was achieved using the following gradient program: 0–15 min, 97% B; 15-16 min, 97–85% B; 16–45 min, 85–50% B; 45–80 min, 50% B. The flow rate was set at 1.0 mL/min and the sample injection volume was 10 *μ*L. The detection wavelength was at 273 nm for gallic acid and 430 nm for curcumin. Data analysis was performed by Similarity Evaluation System for Chromatographic Fingerprint of Traditional Chinese Medicine composed by Chinese Pharmacopoeia Committee (Version 2009 A).

### 2.2. Animals and Drugs

Sprague-Dawley male rats (*n* = 40), weighing 180~220 g, were purchased from the animal center in Tongji Medical College of Huazhong University of Science & Technology. All animals were housed at 22 ± 2°C, 45–75% relative humidity, and 12 h light-dark cycles. They were all able to freely access food and water and get 1-week acclimation to the new environment before the experiment. All the experimental procedures were performed following the International Guidelines for Care and Use of Laboratory Animals and approved by the Animal Ethical Committee of the Institute of Health and Epidemic Prevention (Wuhan, China). Tang-Kang-Fu-San (TKFS) was provided by Tibet Autonomous Region Institute of Traditional Tibetan Hospital (Lhasa, China). Metformin tablets were purchased from Beijing Jing Feng Pharmaceutical Factory (Beijing, China).

### 2.3. Induction of Type 2 Diabetes Mellitus (T2DM)

In a total of 40 rats, 6 of them were randomly assigned to the normal control group and fed with standard laboratory chow. The other 34 rats were assigned to other experimental groups to induce T2DM by a low-dose STZ and high-fat diet as described [12]. Briefly, the rats were fed with high-fat diet containing 20% carbohydrate, 20% protein, and 60% fat (Research Diets Inc., New Jersey, USA) for 4 weeks followed by an intraperitoneal (i.p.) injection of STZ (35 mg/kg). 72 hours after STZ injection, the fast blood glucose in rats was measured and only those rats with plasma glucose levels ≥11.1 mmol/L (referred to as hyperglycemia) were used for next experiments. These 34 rats were fed with high-fat diet throughout the whole study, and among them 1 rat unfortunately died and 3 rats failed to develop hyperglycemia.

### 2.4. Experimental Design

T2DM rats were randomly divided into five groups (*n* = 6 per group) as follows: T2DM rats treated with vehicle (0.9% saline), T2DM rats treated with 200 mg/kg metformin, and T2DM rats treated with 0.3 g/kg, 0.6 g/kg, and 1.2 g/kg TKFS. The doses of TKFS above were used for the rats because we converted the human doses to rat-equivalent doses with scaling by body surface area, according to instructions from that used in human beings (6 g/day per adult). Both TKFS and metformin were dissolved in 0.9% saline, and rats were treated by oral gavage administration with doses described above once a day for 3 weeks. Among these 5 groups and normal control group animals, their body weight and blood glucose were checked weekly during the study. At day 18, their oral glucose tolerance test (OGTT) was performed. At the end of day 21, all rats were anesthetized with pentobarbital (40 mg/kg body weight, i.p.) and their blood was quickly collected from the hearts and centrifuged at 4°C and 3000 r/min for 15 min and the serum was collected and stored at −20°C for biochemical analysis. The pancreas tissues were dissected and immediately immersed in 4% paraformaldehyde for histological analysis. The hindlimb skeletal muscle was dissected and immediately frozen in liquid nitrogen for western blot analysis.

### 2.5. Fasting Blood Glucose (FBG) and Body Weight

FBG was measured in a drop of blood from the tails by glucose oxidase method using a glucometer (OneTouch, Ultra, LifeScan, USA) once a week for three weeks (after plasma glucose levels ≥11.1 mmol/L). Their body weight was measured with an electronic weighing scale.

### 2.6. Oral Glucose Tolerance Test (OGTT)

For the OGTT, the rats of all groups were fasted overnight and then orally loaded with glucose (2 g/kg). 0, 30, 60, and 120 min after glucose administration blood samples were collected via the tail veins and glucose levels were measured. The total area-under-the-curve (AUC) of glucose of the sampling period from 0 to 120 minutes was determined by the following formula: the AUC value = 1/4 × (BG0) + 1/2 × (BG30) + 3/4 × (BG60) + 1/2 × (BG120) [13].

### 2.7. Fasting Serum Insulin Levels (FINS) and Homeostasis Model Assessment of Insulin Resistance (HOMA-IR) Analysis

FINS were measured by using an enzyme-linked immunosorbent assay insulin ELISA kit (CSB-E05070r, CUSABIO BIOTECH Co., Ltd, Wuhan, China). Homeostasis Model Assessment-Insulin Resistance (HOMA-IR) was applied to assess insulin resistance by the following formula: HOMA score = fasting serum insulin (*μ*U/mL) × fasting glucose (mmol/L)/22.5 [13, 14]. After logarithmic transformation the statistical analysis was performed.

### 2.8. Biochemical Analysis

The total cholesterol (TC), triglycerides (TG), LDL cholesterol (LDL-C), and HDL cholesterol (HDL-C) levels in rat blood serum were determined with absorption spectrophotometric analysis by an automatic biochemical analyzer (Hitachi 7180 + ISE, Tokyo, Japan). All results were analyzed and reported by the machine automatically.

### 2.9. Histological Analysis of Pancreas Tissues

The pancreas tissues were dissected and then fixed in 4% paraformaldehyde overnight followed by dehydration and paraffin embedding. The paraffin-embedded tissue specimens were sliced into 4 *μ*m thickness sections, which were mounted on glass slides and stained with Hematoxylin and Eosin (HE) staining and examined with a light microscopy (Olympus, Tokyo, Japan) for histological analysis.

### 2.10. Western Blot Analysis

The total protein of the skeletal muscle was extracted as described previously [15]. Next the protein was fractionated on 10% SDS-PAGE and transferred onto PVDF membrane. After blocking with 5% nonfat milk for 1 hour, the membranes were incubated at 4°C overnight with primary antibodies: anti-AKT (#4685), anti-phospho-AKTSer473 (#4060), anti-GLUT4 (#2213) (CST, Massachusetts, USA), and anti-GAPDH (Proteintech, Wuhan, China) diluted at 1 : 1000. Then the membranes were washed in TBST and incubated with appropriate horseradish peroxidase-conjugated secondary antibodies. The protein bands were visualized by using a BeyoECL Plus (P0018, Beyotime Biotechnology, China), and a densitometry analysis was performed by BioRad Quantity One software [13]. GAPDH was used as the internal control for semiquantitative analysis.

### 2.11. Statistical Analysis

All data were expressed as the means ± SEM. GraphPad Prism 5 software was used for the data statistical analysis and graphics. Unpaired* t*-test was used to analyze statistical comparisons between two groups. Multiple comparisons statistical significance were compared by one-way ANOVA. *P* value < 0.05 was assumed as statistically significant.

## 3. Results

### 3.1. The Contents of Gallic Acid and Curcumin in TKFS

**Figure 1:**
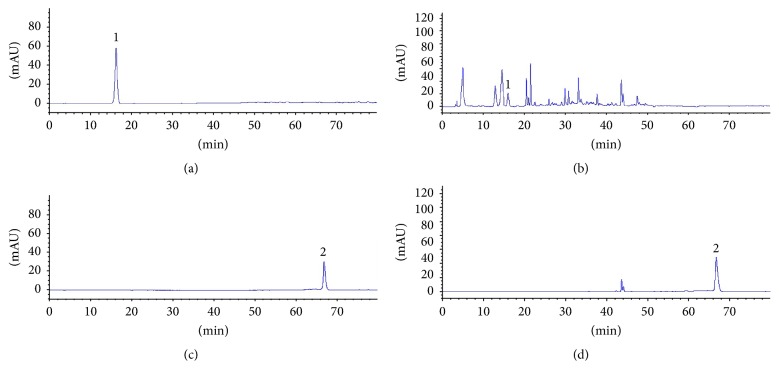
The HPLC chromatogram of TKFS sample and reference substance at different detection wavelength ((a) gallic acid at 273 nm, (b) TKFS sample at 273 nm, (c) curcumin at 430 nm, and (d) TKFS sample at 430 nm).

TKFS mainly consists of* Berberis kansuensis *Schneid.,* Curcuma longa *L.,* Phyllanthus emblica,* and so forth of 11 medicinal herbs (from the product labels and data not shown). Our developed method successfully determined their features, especially those identities of gallic acid and curcumin in TKFS (Figure 1). Contents of the two compounds in the samples are 0.16% and 0.24%. In the present study, since T2DM rats were treated with 0.3 g/kg, 0.6 g/kg, and 1.2 g/kg TKFS, the doses administered presented in the herbal formula with gallic acid are 0.48 mg/kg, 0.96 mg/kg, and 1.92 mg/kg and with curcumin are 0.72 mg/kg, 1.44 mg/kg, and 2.88 mg/kg, respectively. The HPLC results showed that the linear ranges of gallic acid and curcumin were 7.10–63.86 *μ*g/mL (*r* = 0.9998) and 5.24–47.40 *μ*g/mL (*r* = 0.9996), respectively. The average recoveries were 98.41% with RSD of 2.04% (*n* = 6) for gallic acid and 99.01% with RSD of 1.85% (*n* = 6) for curcumin. It was concluded that the HPLC method was simple and accurate with high sensitivity and good repeatability which can be used for the determination of phytochemical features of multiherbal formulae TKFS.

### 3.2. Effects of TKFS on Fasting Blood Glucose (FBG) and Body Weight

**Figure 2:**
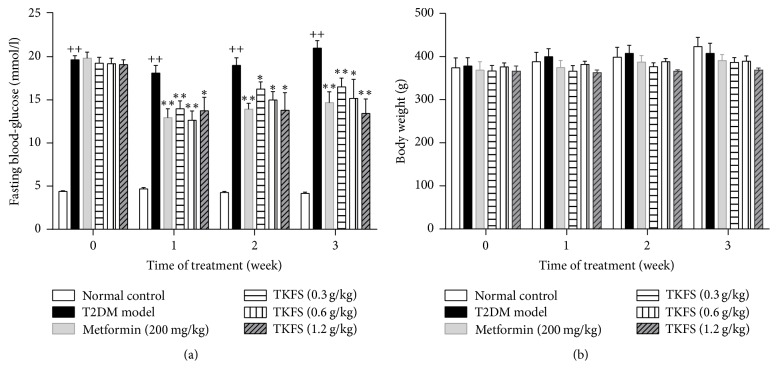
Effects of TKFS on FBG (a) and body weight (b) in T2DM rats. ^++^*P* < 0.01 versus normal control group; ^*∗*^*P* < 0.05 and  ^*∗∗*^*P* < 0.01 versus T2DM model group. Results are presented as means ± SEM (*n* = 6 each group).

The levels of FBG were higher in the rats of the diabetic model control group than those of the normal control group during all weeks we studied (all *P* < 0.01). After treatment with all different doses of TKFS, the levels of FBG were significantly decreased compared with T2DM rats treated with vehicle (*P* < 0.05 or *P* < 0.01) (Figure 2(a)). However, there was no significant difference in relative body weight among all T2DM mice no matter treated with different doses of TKFS, metformin, or vehicle (*P* > 0.05) (Figure 2(b)).

### 3.3. Effects of TKFS on OGTT

**Figure 3:**
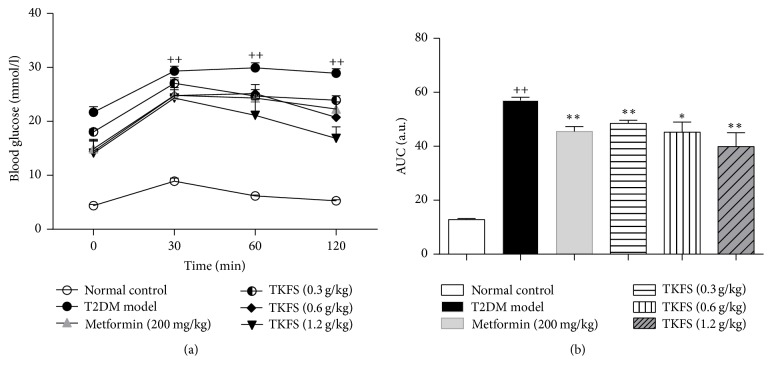
Effects of TKFS on OGTT in T2DM rats. ^++^*P* < 0.01 versus normal control group; ^*∗*^*P* < 0.05 and  ^*∗∗*^*P* < 0.01 versus T2DM model group. Results are presented as means ± SEM (*n* = 6 each group).

Similar to FBG results, blood glucose levels tested by OGTT in the T2DM rats treated with vehicle were higher than those of the normal control group at all time points after oral glucose administration (*P* < 0.01) (Figure 3(a)). From the AUC of the five groups, we could observe an obvious reverse in blood glucose levels in the TKFS- and metformin-treated groups compared with T2DM rats treated with vehicle (*P* < 0.05 or *P* < 0.01) (Figure 3(b)).

### 3.4. The Effect of TKFS on FINS and HOMA-IR Index

**Figure 4:**
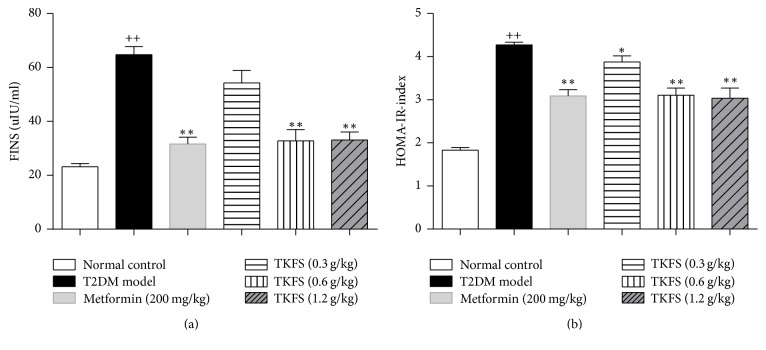
Effects of TKFS on FINS (a) and HOMA-IR (b) index in T2DM rats. ^++^*P* < 0.01 versus normal control group; ^*∗*^*P* < 0.05 and ^*∗∗*^*P* < 0.01 versus T2DM model group. Results are presented as means ± SEM (*n* = 6 each group).

In T2DM rats, dramatic increases in serum fasting blood insulin and HOMA-IR index levels were observed. As shown in Figures 4(a) and 4(b), both of them were decreased after treatment of TKFS at 0.6 g/kg and 1.2 g/kg compared to vehicle treatment. TKFS at 0.3 g/kg did not cause significant changes in the FINS as compared with their vehicle controls in T2DM rats (Figure 4(a)), but it did in the HOMA-IR index significantly (Figure 4(b)).

### 3.5. Effects of TKFS on Plasma Lipid Parameters

**Figure 5:**
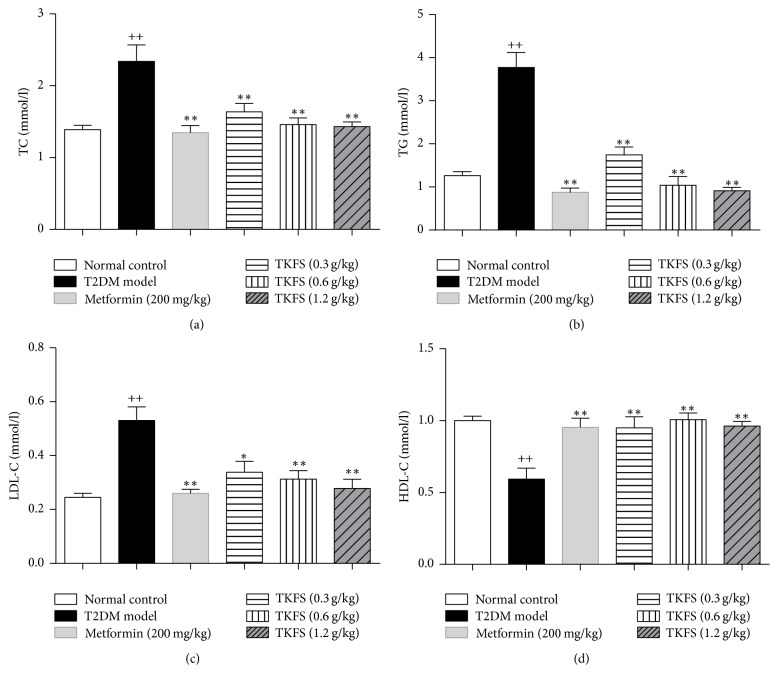
Effects of TKFS on TC, TG, LDL-C, and HDL-C in T2DM rats. TC (a), TG (b), LDL-C (c), and HDL-C (d); ^++^*P* < 0.01 versus normal control group; ^*∗*^*P* < 0.05 and  ^*∗∗*^*P* < 0.01 versus T2DM model group. Results are presented as means ± SEM (*n* = 6 each group).

Dyslipidemia was developed evidently in our T2DM rats as lipid profile (TC, TG, and LDL-C) in serum was significantly elevated in T2DM rats treated with vehicle compared to normal control group (*P* < 0.01). TKFS- and metformin-treated groups significantly reversed the rise in the levels of serum TC, TG, and LDL-C in experimental T2DM rats (Figures 5(a)–5(c)). Moreover, in T2DM rats, decreased level of serum HDL-C was observed, and after treatment with TKFS (0.3 g/kg, 0.6 g/kg, and 1.2 g/kg) for 21 days, it was significantly increased (*P* < 0.01) (Figure 5(d)).

### 3.6. Effects of TKFS on the Pathomorphism of Pancreas Tissues

**Figure 6:**
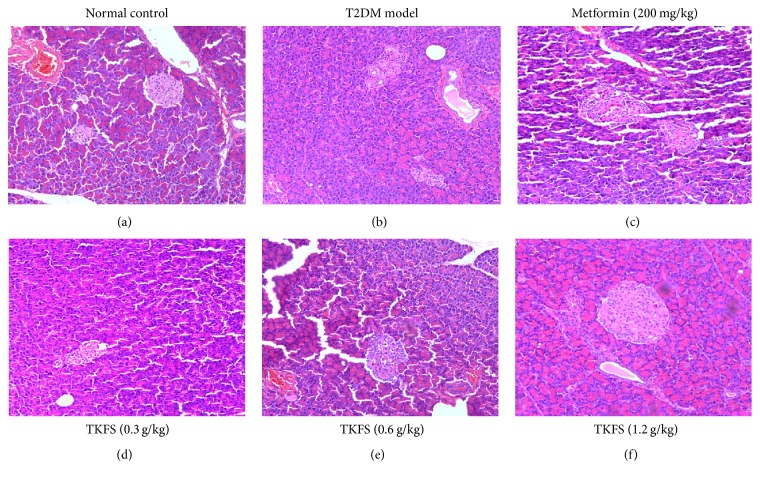
Effects of TKFS on the pathomorphism of pancreas tissues in T2DM rats. Representative H&E staining images in pancreas paraffin sections of different groups as marked above or below the pictures, respectively. Original magnification ×200.

Compared to the normal control rats (Figure 6(a)), the T2DM rats (T2DM model control group, Figure 6(b)) showed more frequently pathological changes, such as atrophy of islet, islet cells necrosis, lacking organization of islet cells, vanished pancreas and pancreatic acini boundaries, or hypertrophy islet cells. Such histological changes were significantly alleviated in the TKFS (Figures 6(d), 6(e), and 6(f)) and metformin (Figure 6(c)) treatment groups compared to the T2DM model control group.

### 3.7. The Effect of TKFS on p-AKT and GLUT4 Expression in Skeletal Muscle

**Figure 7:**
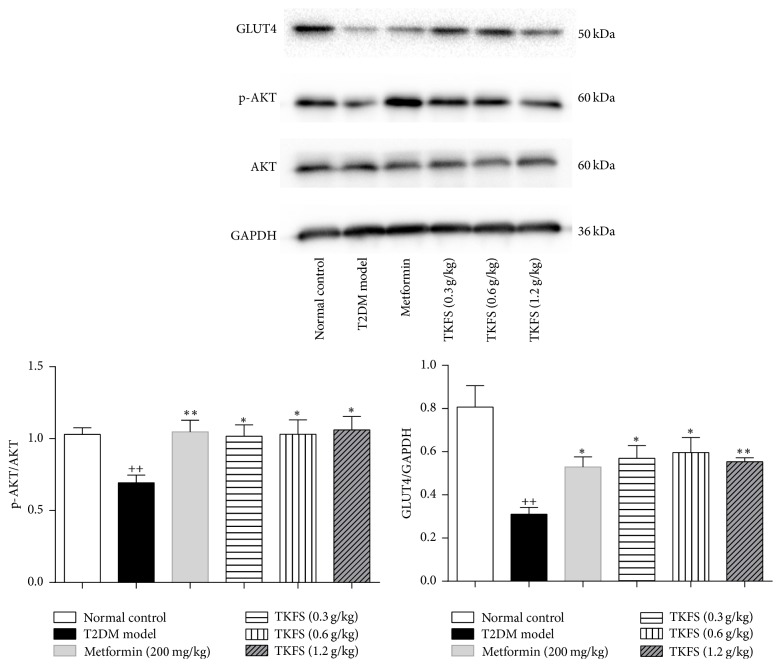
Effects of TKFS on p-AKT^Ser473^ and GLUT4 in skeletal muscle in T2DM rats by western blot analysis. ^++^*P* < 0.01 versus normal control group; ^*∗*^*P* < 0.05 and  ^*∗∗*^*P* < 0.01 versus T2DM model group. Results are presented as means ± SEM (*n* = 6 each group).

To investigate whether intracellular PI3K/AKT pathway or GLUT4 on the cell membranes might be responsible for the antidiabetic effects of TKFS, protein levels in skeletal muscle were assessed by determining GLUT4 and the phosphorylated form of AKT (p-AKT). As shown in Figure 7, the protein expression of p-AKT and GLUT4 is significantly reduced in T2DM rats treated with vehicle compared with that in normal control group (*P* < 0.01). Administration of TKFS at the three doses or metformin for 3 weeks was able to reverse the downregulation of p-AKT (*P* < 0.05 at all three doses) and GLUT4 expression in skeletal muscle (*P* < 0.01 at TKFS at 1.2 g/kg and *P* < 0.05 at the other two doses).

## 4. Discussion

T2DM, the most common form of DM in diabetic patients worldwide, is characterized by hyperglycemia, hyperlipidemia, and insulin resistance [1, 16]. A combined treatment with the high-fat diet and low-dose STZ caused insulin-resistant condition in rats and an elevation of plasma lipid [17, 18], which developed a pathogenesis and process in the T2DM rats perfectly resembling those in human T2DM disease. Therefore we used the high-fat diet combined with STZ-induced T2DM rats for this study.

Before animal work, from 13 chemical constituents in TKFS we quantitatively characterized two of them, gallic acid and curcumin (Figure 1), which turned out to be a remarkable concurrence of their roles in antidiabetes as indicated before. Gallic acid is a major component of extracts from natural fruits or flower and so forth, for effective type 2 diabetes prevention and treatment [19, 20]. In the last decades more than 7000 research publications had revealed the various perspectives of curcumin, such as anti-inflammation, antioxidant, and anticancer activities, as well as being famous for reversing insulin resistance and hyperglycemia and alleviating diabetic associated complications [21–23].

In our* in vivo* studies, we attempted to study antidiabetic effects and the possible intracellular mechanisms of TKFS in treating T2DM. To this end, we tested this agent in rats with STZ-induced T2DM. In our study, high-fat diet fed-STZ-induced diabetic rats developed a stably increased fasting blood glucose and evident insulin resistance. After treatment with TKFS for 3 weeks, characteristic indices including fasting blood glucose (Figure 2(a)), blood glucose following OGTT and AUC of OGTT blood glucose (Figure 3), and FINS and HOMA-IR index (Figure 4) were decreased significantly in TKFS treated diabetic rats compared with diabetic control rats. Therefore, TKFS can decrease both blood glucose and insulin resistance in diabetes. Our results also confirmed that the TKFS showed a strong hypolipidemic effect as well as hypoglycemic effect through the reduction of TG, TC, LDL-C, and elevation of HDL-C (Figure 5). Additionally similar to metformin, TKFS significantly rescued the morphological abnormalities, which provided another evidence that TKFS is capable of protecting pancreatic *β*-cells (Figure 6).

The reports of metformin on reduction in body weight gain are varied, depending on experimental animals, the doses and length of time used, and so forth. Note that in the present study, metformin treatment (200 mg/kg for 3 weeks) did not have obvious effects on reduction of the body weight gain, consistent with one of our previous studies [12] and other similar studies on mice [24, 25]. The “neutral” effect of TFKS on body weight is rather surprising (Figure 2(b)); however, considering that a relatively large proportion of T2DM patients are not obese at all particularly in Asian countries [26], TKFS could be an adequate therapeutic for the population of not obese T2DM patients.

To examine the possible mechanisms for TKFS playing its therapeutic role, we measured p-AKT and GLUT4 levels in skeletal muscle, since the activation of intracellular PI3K/AKT pathway could excite translocation of GLUT4 enhancing glucose uptake and utilization [27, 28]. Downregulated p-AKT and the reduction in GLUT4 expression were detected in skeletal muscle in T2DM model control rats; however both of them were significantly restored with TKFS treatment (Figure 7). These observations indicated that promoting of p-AKT and GLUT4 activities is at least in part responsible for TFKS exerting its effects on opposing hyperglycemia and insulin resistance. Our data is very consistent with a previous study performed in STZ-diabetic rats treated with curcumin in yoghurt, which proved curcumin-enriched yoghurt may promote a direct AKT activation and further increase the GLUT4 translocation in skeletal muscle [21]. Furthermore, such cohorts also strongly support those nutraceutical compounds like curcumin or multiherbal formulae like TKFS exert their effects through various molecular targets and treat various diseases for anti-infectious, anti-inflammatory, and even anticarcinogenic activities [21–23, 29], which may be of their potential advantages compared to those well-known antidiabetics like metformin.

## 5. Conclusion

In the present study, we quantitatively analyzed the active constituents of gallic acid and curcumin in TKFS. We also found TKFS could effectively alleviate diabetic syndromes, maintain the glucose homeostasis, and protect against insulin resistance in high-fat diet and low-dose STZ-induced T2DM rats. The underlying molecular mechanisms are at least through activation of intracellular PI3K/AKT pathway and promoting the translocation of GLUT4 in skeletal muscle.
